# Exfoliation and Characterization of V_2_Se_9_ Atomic Crystals

**DOI:** 10.3390/nano8090737

**Published:** 2018-09-18

**Authors:** Bum Jun Kim, Byung Joo Jeong, Seungbae OH, Sudong Chae, Kyung Hwan Choi, Tuqeer Nasir, Sang Hoon Lee, Kwan-Woo Kim, Hyung Kyu Lim, Ik Jun Choi, Ji-Yun Moon, Hak Ki Yu, Jae-Hyun Lee, Jae-Young Choi

**Affiliations:** 1 SKKU Advanced Institute of Nanotechnology, Sungkyunkwan University, Suwon 16419, Korea; kbj454@skku.edu (B.J.K.); chhcc12@gmail.com (K.H.C.); tuqeernasir166@gmail.com (T.N.); 2 School of Advanced Materials Science and Engineering, Sungkyunkwan University, Suwon 16419, Korea; jbj929@skku.edu (B.J.J.); nysbo0219@gmail.com (S.O.); csd5432@gmail.com (S.C.); alfhdj@gmail.com (S.H.L.); rhksdn8904@gmail.com (K.-W.K.); hyungkyu1992@gmail.com (H.K.L.); cksoon16@gmail.com (I.J.C.); 3 Department of Materials Science and Engineering and Department of Energy Systems Research, Ajou University, Suwon 16499, Korea; ydnas96@ajou.ac.kr (J.-Y.M.); hakkiyu@ajou.ac.kr (H.K.Y.)

**Keywords:** V_2_Se_9_, atomic crystal, mechanical exfoliation, scanning Kelvin probe microscopy

## Abstract

Mass production of one-dimensional, V_2_Se_9_ crystals, was successfully synthesized using the solid-state reaction of vanadium and selenium. Through the mechanical exfoliation method, the bulk V_2_Se_9_ crystal was easily separated to nanoribbon structure and we have confirmed that as-grown V_2_Se_9_ crystals consist of innumerable single V_2_Se_9_ chains linked by van der Waals interaction. The exfoliated V_2_Se_9_ flakes can be controlled thickness by the repeated-peeling method. In addition, atomic thick nanoribbon structure of V_2_Se_9_ was also obtained on a 300 nm SiO_2_/Si substrate. Scanning Kelvin probe microscopy analysis was used to explore the variation of work function depending on the thickness of V_2_Se_9_ flakes. We believe that these observations will be of great help in selecting suitable metal contacts for V_2_Se_9_ and that a V_2_Se_9_ crystal is expected to have an important role in future nano-electronic devices.

## 1. Introduction

To overcome the high-density integration of electronic technology, which faces physical limitations (e.g., fabrication process and reduction in charge carrier mobility), researchers have been intensively trying to develop a new device architecture or novel materials [1,2,3,4]. A range of diverse candidate materials have been proposed since the 2000s. Among them, graphene, which is a single layer of carbon atoms arranged in a hexagonal lattice, is considered to be a promising solution for future electronic devices because of its superior physical properties such as high carrier mobility and excellent chemical stability; however, it has the fatal disadvantage in that it has difficulty forming a band gap [1,2,5,6,7]. Therefore, the development of applications for graphene-based electronic devices, the most promising field, does not meet public’ expectation yet. Graphene nanoribbons (GNRs) are presented as the effective way to open the bandgap of graphene but it is difficult to produce a uniform width in large area [6,7]. In addition, the transport behavior of GNRs and newly introduced two-dimensional (2D) materials (e.g., transition metal dichalcogenides (TMDCs) and black phosphorous), with appropriate bandgaps, are reduced dramatically because of dangling bonds at the side edges and domain boundaries [8,9,10,11]. Unfortunately, most of the studies of the 2D material-based electronic devices thus far contain an etching process to define the conducting channel. Thus, the discovery of one-dimensional (1D) nanomaterials, which are free from edge and grain boundary scattering, is a key solution in the development of nano-electronic device.

Carbon nanotubes (CNTs), which exhibit high carrier mobility, ultimate mechanical strength, and chemical stability, have been considered as representative building blocks for next-generation transistors, chemical sensors, and nanocomposites [12,13,14]. However, the wide range of electronic structures that arise from the different chirality of the CNTs curtails the reliability of the manufacturing process of the nano-electronic devices [15]. Therefore, separation of single-chirality CNTs from the bulk CNTs or control of the chirality during the growth of the CNTs is required. Recently, studies on the synthesis and application of a new family of 1D nanomaterials in the form of three-dimensional (3D) bundles of numerous single-molecular chains coupled by weak van der Waals interactions have been reported [16,17,18,19,20,21]. For example, extensive studies on bulk synthesis and atomic-scale dispersion of the bio-compatible Mo_6_S_9−x_I_x_ have been reported [21,22,23]. In addition, Sb_2_S_3_ was developed as an optoelectronic device by effectively reducing exciton decay due to the absence of dangling bonds [24]. Moreover, VS_4_ was utilized for an electrochemical energy storage device by using the van der Waals gap between the chains [25,26]. However, in the majority of studies on these materials, they have been utilized only as a thin-film structure, although the benefits of the layered characteristics can be exploited. In addition, the crystal structure of Mo_6_S_9−x_I_x_ is not well defined because the position of the sulfur and iodine atoms bridged to the molybdenum atoms may vary even for the same stoichiometric composition.

In this study, we succeeded in mass producing 1D semiconductor V_2_Se_9_ crystals via a simple transport method. Through the mechanical exfoliation method, we confirmed that as-grown V_2_Se_9_ crystals consist of innumerable single V_2_Se_9_ chains linked via the van der Waals interaction, like graphite. In addition, a nanoribbons structure of V_2_Se_9_ which is capable of thickness control was obtained through repetitive mechanical exfoliation of the V_2_Se_9_ crystals. Lastly, the change in work function according to the thickness change of the V_2_Se_9_ flakes was analyzed by scanning Kelvin probe microscopy (SKPM) measurement.

## 2. Materials and Methods

**Synthesis:** V_2_Se_9_ was synthesized using V (Powder, −325 mesh, 99.5%, Sigma-Aldrich, St. Louis, MO, USA) and Se (powder, 99+%, Alfa Aesar, Haverhill, MA, USA). The mixture of V (0.2038 g) and Se (1.4213 or 1.9898 g) with a V to Se ratio of 2:9 or 2:12.6 was pelletized and then sealed in a 10 cm-long evacuated quartz tube. The quartz ampoule was heated for 120 h at a temperature of 300–400 °C (at 5.5 °C/h) and then cooled (at 10 °C/h). The resulting material was a dark gray sintered powder. The unreacted Se was sublimated by heat treatment in a tube furnace at 250 °C under Ar atmosphere for 24 h.

**Mechanical exfoliation:** The bulk V_2_Se_9_ was placed on wafer dicing tape (BT150EKL, Nitto Denko, Umeda, Osaka, Japan) and the materials were stuck several times to yield thinner-than-bulk materials. A substrate (300 nm SiO_2_/Si or bare Si) was cleaned by ultrasonication in acetone, ethanol, and DI water for 15 min, followed by heating at 100 °C in order to remove the moisture from the substrate. The polymer tape was adhered strongly to and pressed against the substrate. After adhesion, the polymer tape was removed from the substrate; this process was repeated for exfoliation.

**Characterization:** Powder X-ray diffraction (Mac Science, M18XHF22, Tokyo, Japan) was performed using Cu-Kα radiation (λ = 0.154 nm). Field emission-scanning electron microscopy (FE-SEM, Hitachi, S4300SE, Chiyoda, Tokyo, Japan) was operated at an acceleration voltage of 15 kV. Atomic force microscopy (AFM, Park systems, NX 10, Suwon, South Korea) was performed in a non-contact mode for the topographic analysis of the mechanically exfoliated V_2_Se_9_ on 300 nm Si/SiO_2_. The surface potentials of V_2_Se_9_ on Si substrate were measured by SKPM (Park systems, NX10, Suwon, South Korea) measurement using Si tips coated with Cr-Pt (Multi75-G, Budget Sensors Inc., 1113 Sofia, Bulgaria) with resonance frequencies of 75 kHz, a scan rate of 0.3 Hz, and sample bias of ±1 V.

## 3. Results and Discussion

**Figure 1:**
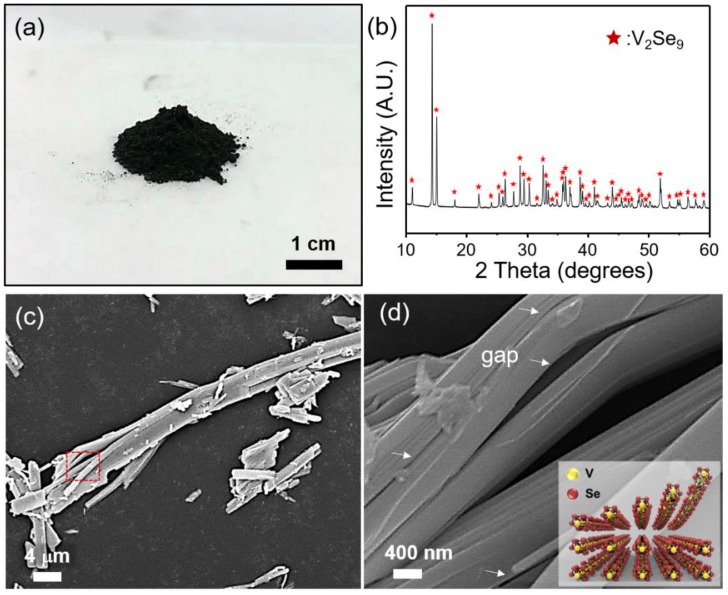
(**a**) Photo-image of mass production of V_2_Se_9_ crystal. (**b**) XRD pattern of V_2_Se_9_ crystal. (**c**) Low- and (**d**) high-magnification SEM images of V_2_Se_9_ crystal. The inset shows an illustration of the crystal structure of V_2_Se_9_.

Since the transition metal vanadium has the outermost 3d orbital, it can produce various forms of compounds (e.g., V_5_Se_4_ to V_2_Se_9_) through a chemical reaction with selenium (see the phase diagram in Figure S1). Therefore, to synthesize V_2_Se_9_ crystals with a high-purity and high-crystallinity, the ratio of V:Se and the synthesis temperature should be considered carefully. For example, if the atomic mixing ratio of V and Se powder is adjusted precisely to 2:9 to synthesize V_2_Se_9_ crystals, unpredictable fluctuation occurs in the synthetic tube and VSe_2_, which is an undesirable impurity, is formed. We corrected these parameters experimentally, and as a result obtained pure V_2_Se_9_ crystals with an exact stoichiometry ratio of 2:9 by adding them in excess of Se, as shown in Figure 1a (V:Se atomic mixing ratio of 2:12.6). The crystallinity of the bulk V_2_Se_9_ crystal was verified by the X-ray diffraction (XRD) pattern (JCPDS 01-077-1589) (Figure 1b). The SEM images in Figure 1c,d clearly shows the 1D nanowire structures and the gaps generated during transfer of the sample onto the Si substrate.

**Figure 2:**
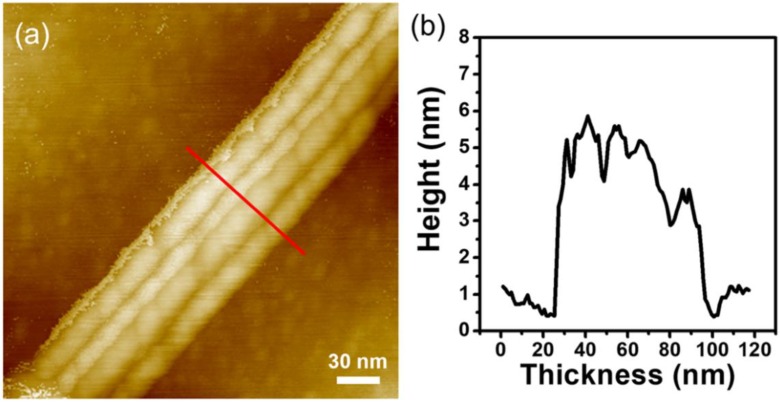
(**a**) Atomic force microscopy (AFM) image of the 1D V_2_Se_9_ flake on 300 nm SiO_2_/Si substrate. (**b**) Line-profile of a V_2_Se_9_ flake as marked in Figure 2a.

To investigate the structural characteristics of nanoscale V_2_Se_9_, the bulk V_2_Se_9_ crystal was mechanically exfoliated using the well-known tape method [1]. Although each single V_2_Se_9_ chains are linked by weak van der Waals interaction, we obtained a thin V_2_Se_9_ nanoribbon on a 300 nm SiO_2_/Si substrate (see in Figure 2). Unlike typical 2D materials, an exfoliated V_2_Se_9_ nanoribbon shows a rough surface.

**Figure 3:**
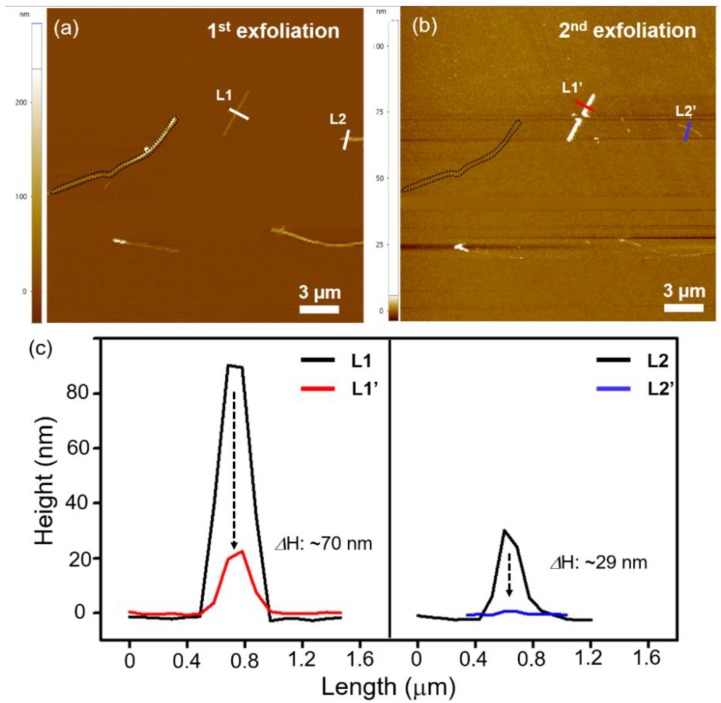
(**a**) AFM image of exfoliated V_2_Se_9_ on 300 nm SiO_2_/Si substrate. (**b**) AFM image of additionally exfoliated V_2_Se_9_ on 300 nm SiO_2_/Si substrate. (**c**) Line-profile of 1D V_2_Se_9_ flakes on 300 nm SiO_2_/Si substrate before and after 2nd exfoliation.

We attempted a further delamination at the sample position using the tape, and found that some of them had been torn out (black dotted line) and that the thickness decreased from 90 to 20 nm (L1 to L1′), and from 31 to 2 nm (L2 to L2′) (see Figure 3).

**Figure 4:**
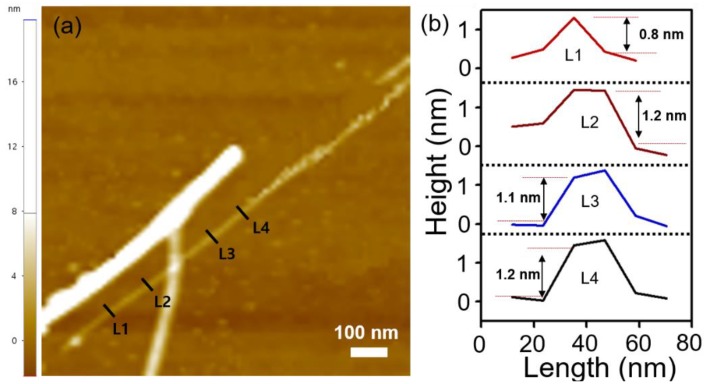
(**a**) AFM image of the V_2_Se_9_ nanoribbon on the 300 nm SiO_2_/Si substrate. The inset shows an illustration of the V_2_Se_9_ nanoribbon. (**b**) Line-profiles of the V_2_Se_9_ nanoribbon as marked L1, L2, L3, and L4 in Figure 4a.

Figure 4a shows the AFM image of an isolated V_2_Se_9_ nanoribbon on the 300 nm SiO_2_/Si substrate. The nanoribbon has an atomic scale thickness and a width of approximately 20 nm (Figure 4b). Since V_2_Se_9_ has a bundle structure in which single chains are bonded by van der Waals forces, we expect that V_2_Se_9_ nanoribbons may exhibit ideal transport characteristics without degradation due to edge scattering.

To investigate the electrical properties of V_2_Se_9_ flakes with a different number of layers, we performed an SKPM analysis, which is a non-destructive analytical tool that can investigate the local surface potential energy and work function by measuring the contact potential difference between the tip and the sample (*V_CPD_*) [27,28]. Because the V_2_Se_9_ nanoribbons were on the bare Si substrate, the work function of V_2_Se_9_ flakes can be calculated using the following equation:

(1) $$V_{CPD}=\frac{1}{e}\left(\varphi_{t}-\varphi_{f}\right),$$

(2) $$\Delta V_{CPD}=V_{CPD}\left(V_{2}Se_{9}\right)-V_{CPD}\left(substrate\right)\;=\frac{1}{e}\left(\varphi_{t}-\varphi_{f}\right)-\frac{1}{e}\left(\varphi_{t}-\varphi_{s}\right)\;=\frac{1}{e}\left(\varphi_{s}-\varphi_{f}\right)$$

where *φ_t_*, *φ_s_* and *φ_f_* represent the work functions of the tip, Si substrate, and V_2_Se_9_ flake, respectively.

**Figure 5:**
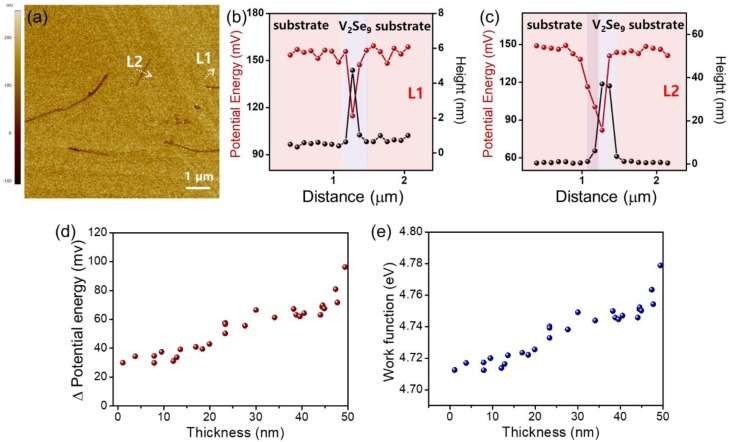
(**a**) Scanning Kelvin probe microscopy (SKPM) image of exfoliated 1D V_2_Se_9_ flakes on the Si substrate. (**b**,**c**) Height and potential energy profiles of the V_2_Se_9_ flakes and Si substrate as labeled in Figure 5a. (**d**,**e**) Variation in potential energy difference and work function depending as a function of thickness of V_2_Se_9_ flakes.

As shown in Figure 5a, the surface potential energy varies with the thickness of the V_2_Se_9_ flakes. For example, the surface potential energy differences (Δ potential energy) between the V_2_Se_9_ flakes with thicknesses of 5 and 40 nm and the Si substrate were 38 and 60 mV, respectively (Figure 5b,c). A Statistical analysis of more than 27 samples shows that as the thickness of the V_2_Se_9_ flake is less than 25 nm, the surface potential energy difference and the work function become to decrease simultaneously (Figure 5d,e). These phenomena can be explained using an interlayer screening effect, which is also observed in typical 2D materials [27,28,29]. In general, the native Si oxide (e.g., SiO_x_), which forms naturally on the surface of the Si wafer, has a hydrophilic property, which caused a large number of charge-trapping sites owing to the moisture in the air. Therefore, it affected the charge transfer between the V_2_Se_9_ flakes and the Si substrate [27]. Since the effective area of the interlayer screening effects increases with decreasing flake thickness, the surface potential difference and the work function of 25 nm thick V_2_Se_9_ flakes decreased from that of the bulk V_2_Se_9_ (See in Figure S2).

## 4. Conclusions

In conclusion, the mass production of the high-purity and high-crystalline 1D material V_2_Se_9_ crystals was successfully demonstrated using the solid-state reaction of V and Se. Through the mechanical exfoliation method, we confirmed that as-grown V_2_Se_9_ crystals consist of innumerable covalently bonded V_2_Se_9_ chains linked by the van der Waals interaction. In addition, atomic nanoribbons structures of V_2_Se_9_ was obtained on the 300 nm SiO_2_/Si substrate. We used SKPM analysis to investigate the electrical characteristics of V_2_Se_9_ and established that the work function decreased with decreasing thickness of the V_2_Se_9_ flakes owing to the interlayer screening effect. These results will be of great help in selecting suitable metal contacts for V_2_Se_9_; these will have a significant influence on the overall performance. We believe that the 1D semiconductor V_2_Se_9_ crystal is expected to be a new family of 2D materials that will be considered essential in future device applications.

## Supplementary Materials

The following are available online at http://www.mdpi.com/2079-4991/8/9/737/s1, Figure S1: Phase diagram of V-Se binary system, Figure S2: SKPM image of exfoliated 1D V_2_Se_9_ flake.
