# Supplementary material for: Exfoliation and Characterization of V2Se9 Atomic Crystals
Source: Nanomaterials (Basel). 2018 Sep 18;8(9):737. doi: 10.3390/nano8090737 (PMC6163525; doi:10.3390/nano8090737)
Supplement: Supplementary file 1 [file nanomaterials-08-00737-s001.pdf]

nanomaterials

Supplementary Information for

Exfoliation and characterization of V_2Se_9 atomic crystals

By Bum jun Kim, Byung Joo Jeong, Seungbae OH, Sudong Chae, Kyung Hwan Choi, Tuqeer Nasir, Sang Hoon Lee, Kwan-Woo Kim, Hyung Kyu Lim, Ik Jun Choi, Ji-Yun Moon, Hak Ki Yu, Jae-Hyun Lee, and Jae-Young Choi

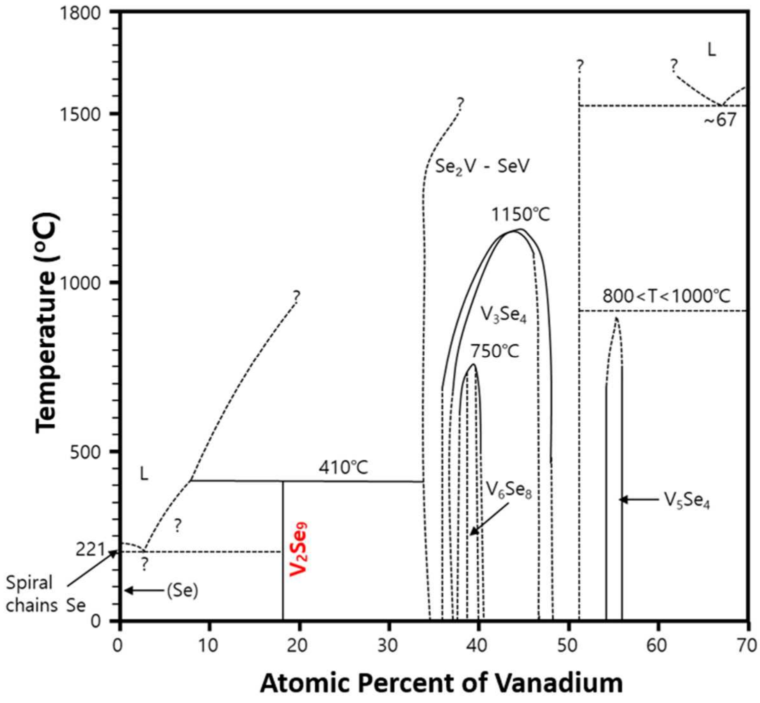

Figure S1. Phase diagram of V-Se binary system [S1].

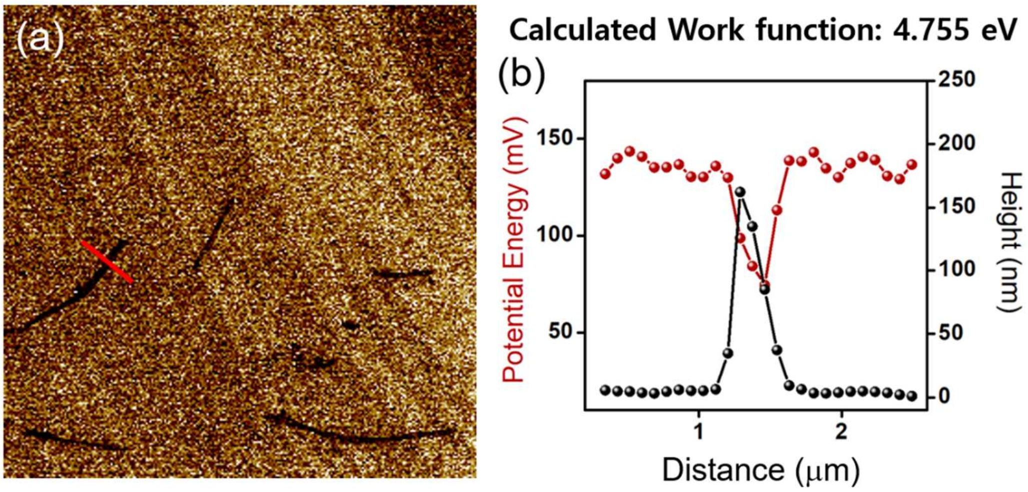

Figure S2. (a) SKPM image of exfoliated 1D V_2Se_9 flakes on the Si substrate. (b) Height and potential energy profiles of the V_2Se_9 flakes and Si substrate as marked in Figure S2a.

References

S1. Massalski, T.B.; Okamoto, H.; Subramanian, P. R.; Kacprzak, L. The Low Temperature Synthesis of Vanadium Selenides Using Superlattice Reactants, *J. Solid State Chem*. **1996**, 123, 337-343.
